# Bone health in patients with inborn errors of metabolism

**DOI:** 10.1007/s11154-018-9460-5

**Published:** 2018-09-12

**Authors:** M. Langeveld, C. E. M. Hollak

**Affiliations:** Department of Endocrinology and Metabolism, Amsterdam UMC, University of Amsterdam, Meibergdreef 9, 1105 AZ Amsterdam, the Netherlands

**Keywords:** Osteopenia, Osteoporosis, Inborn error of metabolism, Lysosomal storage disorder, Skeletal dysplasia, Bone metabolism

## Abstract

Inborn errors of metabolism encompass a wide spectrum of disorders, frequently affecting bone. The most important metabolic disorders that primarily influence calcium or phosphate balance, resulting in skeletal pathology, are hypophosphatemic rickets and hypophosphatasia. Conditions involving bone marrow or affecting skeletal growth and development are mainly the lysosomal storage disorders, in particular the mucopolysaccharidoses. In these disorders skeletal abnormalities are often the presenting symptom and early recognition and intervention improves outcome in many of these diseases. Many disorders of intermediary metabolism may impact bone health as well, resulting in higher frequencies of osteopenia and osteoporosis. In these conditions factors contributing to the reduced bone mineralization can be the disorder itself, the strict dietary treatment, reduced physical activity or sunlight exposure and/or early ovarian failure. Awareness of these primary or secondary bone problems amongst physicians treating patients with inborn errors of metabolism is of importance for optimization bone health and recognition of skeletal complications.

## Introduction

Bone is frequently affected by inborn errors of metabolism. Some disorders primarily affect bone and present with prominent skeletal features such as the mucopolysaccharidoses, while in other disorders the alterations, such as decreased bone mass, may be secondary to nutritional deficiencies as a consequence of strict diets, inflammatory processes, hypogonadism and/or drugs, especially anti-epileptic treatments [1]. Mirroring the range of pathophysiological mechanisms involved, therapeutic approaches will be different for the wide range of disorders. However, some generalizations can be made: in all conditions, physicians should be aware of general measures to optimize bone health such as adequate intake of calcium/phosphate and vitamin D and optimal physical activity. In patients with cognitive dysfunction, the latter can be very challenging. Early identification and management of potential risk factors is essential for skeletal health in adulthood.

**Fig. 1:**
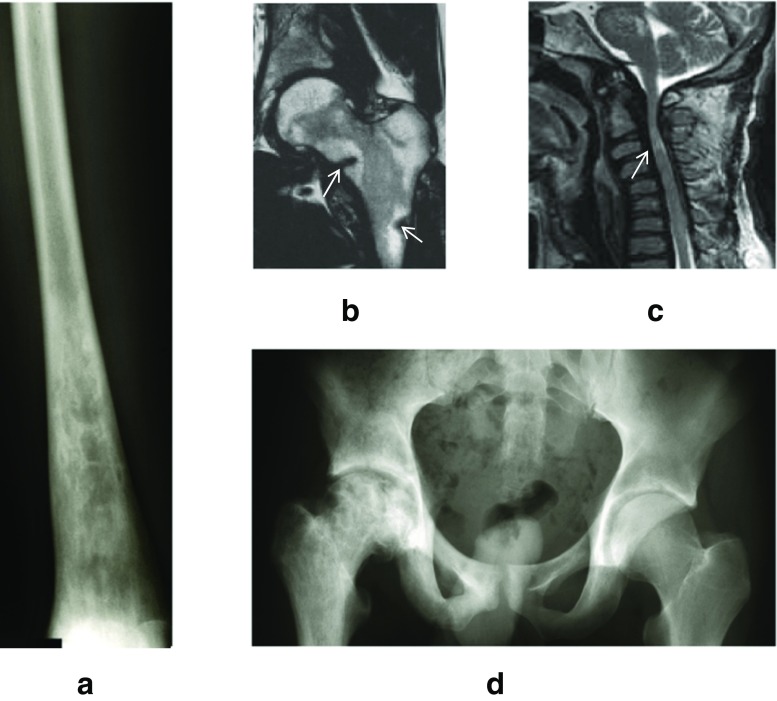
Examples of typical skeletal abnormalities in patients with inborn errors of metabolism. **a**. Erlenmeyer flask deformity in the distal femur of a patient with type 1 Gaucher disease (plain radiograph). **b**. Fragility fractures (arrows) in the femoral neck and diaphysis of a patient with X-linked hypophosphatemic rachitis (T2-MRI). **c**. Myelopathy (arrow) due to narrowing of the spinal canal in MPS-I. Note the platyspondylia (flattened vertebral bodies) as part of the dysostosis multiplex (T2-MRI). **d**. Avascular necrosis on the right side, normal femoral head on the left in a patient with Gaucher disease (plain radiograph)

In this article, we discuss primary and secondary involvement of the bone in inborn errors of metabolism. The first group includes those primarily affecting the bone marrow, mineral component or cartilage, in particular the lysosomal storage disorders, hypophosphatasia and hereditary hypophosphatemic rickets (Table 1, Fig. 1). The second group encompasses mainly disorders of aminoacid metabolism such as phenylketonuria, lysinuric protein intolerance and homocysteinuria (Table 2).

**Table 1:** Inborn errors of metabolism primarily affecting the bone

	Symptoms	Disorder	Additional signs
Inborn errors of calcium and phosphorus homeostasis	Short stature, lower extremity deformities;	Hereditary hypophosphatemic rickets	Fatigue and weakness decreased plasma phosphate
	Perinatal: respiratory distress, hypotonia Older:rachitis-like features, chronic pain, recurrent fractures	Hypophosphatasia	Calcific deposits; decrease plasma bone specific alkaline phosphatase
Involvement of bone marrow	Bone pain, bone crises, pathological fractures, avascular necrosis, osteoporosis	Gaucher disease type 1 or 3;	Hepatosplenomegaly, cytopenia
	Arthritis, osteopenia	Niemann Pick A/B	Hepatosplenomegaly, cytopenia, interstitial lung disease
Mucopolysaccharidoses and mucolipidoses	Dysostosis multiplex Short stature Joint stiffness or laxity (MPS IV)	MPSes and mucolipidosis type II and III	Specific facial features and skull shape, abdominal and inguinal hernias (MPSes), corneal clouding, carpal tunnel syndrome, ENT problems (MPSes), cognitive decline (MPS IH/HS, MPS II, ML II)
Miscellaneous	Osteopetrosis	Pycnodysostosis	Short stature, pathological fractures
	Dysostosis multiplex	Mannosidosis	Psychiatric symptoms, corneal clouding or cataract, hearing loss, immune deficiencies, myopathy.
Progressive deformation of the spine and arthrosis of the large joints		Alkaptonuria	Genitourinary tract stones, cardiac valve disease, dark urine, pigmentation of the auricle and sclera

**Table 2:** Inborn errors of metabolism with secondary bone disease

Skeletal symptom/sign	Disorder	Additional symptoms
Osteoporosis	All inborn errors of metabolism that require strict dietary treatment	Miscellaneous; frequently neurological symptoms
	Galactosemia	Cognitive impairment, primary ovarian failure, cataract*
	Phenylketonuria	Cognitive impairment in untreated patients^#^
	Homocystinuria	Marfanoid habitus, kyphosis, lens luxation, cognitive impairment
Osteoporosis Pathological fractures	Lysinuric Protein Intolerance	Protein avoidance, gastrointestinal symptoms hyperammonaemia, lung disease
	Wilson disease	Liver disease and /or neurological and psychiatric manifestations
	GSD type II (Pompe disease)	Muscle weakness, secondary respiratory impairment

*Cataract in untreated patients only ^#^In patients born before the introduction of newborn screening or in countries were NBS for this disorder is not performed

Finally, diagnostic strategies and general recommendations are provided.

## Primary involvement of the skeleton

### Disorders affecting calcium and/or phosphate homeostasis

Calcium and phosphate are essential components of bone and have important roles in several physiological functions as well. For this reason homeostasis is tightly regulated involving not only the bone but also intestine and kidney. Mutations in several key regulatory genes may affect parathyroid hormone function, calcium sensing or phosphate transporters resulting in a variety of clinical symptoms [2]. The following section will focus be on inherited metabolic bone disorders with an overt skeletal phenotype, i.e. hypophosphatasia and hereditary hypophosphatemic rickets.

### Hypophosphatasia

Hypophosphatasia is caused by low activity of the tissue non-specific isoenzyme of alkaline phosphatase (TNSALP) caused by a mutation in the *ALPL* gene. The enzyme normally degrades extracellular inorganic pyrophosphate (PPi) into Pi (inorganic phosphate). Defects in this metabolic pathway leads to accumulation of PPi in the extracellular bone matrix and low alkaline phosphatase levels. [3] Other substrates of TNSALP are pyridoxal-5-phosphate (PLP), and phosphoethanolamine (PEA). PLP is the biologically active form of vitamin B6 and high levels, as seen in hypophosphatasia, are believed to be involved in neurotoxicity. The pathophysiology of the disorder is complex: increased PPi levels inhibit normal mineralization resulting in rickets and osteomalacia, but other tissues and organs can be affected as well. For example, muscle hypotonia is a well-known feature of the disease [4]. The disorder is extremely variable, ranging from severe infantile phenotypes to almost asymptomatic adult cases, with only dental problems. Most common skeletal symptoms are those of rickets, with bone pain, fractures and bowing of legs. The muscle hypotonia may add to walking problems. In the most severe perinatal cases, hypotonia and respiratory distress can lead to early death. Children can develop craniosynostosis and frequently have retarded growth. In adults, normal height can be achieved, but patients may suffer from skeletal complications such as fragility fractures and chronic pain [5]. Treatment of hypophosphatasia consists of supportive care, including regular periodontal and dental care to avoid inflammation, sufficient physical activity and orthopedic interventions. In adult patients, several other modalities have been tried, including the use of teriparatide (parathyroid hormone amino acid 1–34) which has been shown to improve fracture healing and resolve stress fractures [6].

Recently, subcutaneous asfotase alfa (Strensiq(®)), a first-in-class bone-targeted human recombinant TNSALP replacement therapy, is approved in the EU for long-term therapy in patients with pediatric-onset hypophosphatasia. It was shown that asfotase alfa in this patient group can improve rickets as evidenced by an improvement in radiographically-assessed severity scores at 24 weeks [7]. Furthermore, patients experienced improvements in respiratory function, gross motor function, fine motor function, growth and quality of life [8]. In life-threatening perinatal and infantile hypophosphatasia, asfotase alfa also improved overall survival [9]. However, not all infantile cases have a favorable outcome [10]. Knowledge on long term effectiveness is still scarce and prognostic factors to determine eligibility for treatment insufficiently known. In view of the high costs of this therapy, collaborative efforts are needed to support the decision making whom to treat. This will also become a challenge for the adult patient group, for whom this modality is still under study.

### Hereditary hypophosphatemic rickets

The most common of the hereditary hypophosphatemic rickets is the X-linked form caused by a mutation in the *PHEX* gene, encoding a phosphate-regulating endopeptidase homolog. Disruption of this enzyme results in a rise in FGF23 levels, suppressing transcription of sodium–phosphate co-transporters in the kidney eventually leading to renal phosphate wasting and hypophosphatemia [11]. An autosomal dominant form resulting from gain of function mutations in FGF23 gives rise to a similar phenotype. Elevated FGF23 levels decrease synthesis and increase catabolism of active vitamin D, resulting in low levels. Patients typically have short stature and lower extremity deformities secondary to rickets at an early age, but milder forms exist. Female carriers of the X-linked variant in particular may present late, sometimes with nonspecific bone pain, fatigue, and weakness. At a later age they may have more localized complaints relating to enthesopathy and early arthropathy. Hypophosphatemia is an important diagnostic finding.

An early diagnosis is important to improve growth and prevent complications [12]. Children should be treated with the active form of vitamin D (calcitriol or alfacalcidol) and phosphate [12, 13]. Not all patients tolerate liquid phosphate very well and thus compliance may be a challenge. For older patients, phosphate tablets may be an alternative. Additional treatment with growth hormone has been tried in children, with inconsistent results and potential side effects. A recent trial suggests that some improvement in pre-pubertal short children can be achieved [14]. Treatment of adults is debated: once adult height has been achieved, the indications for therapy are to reduce osteomalacia and related pain symptoms. It may be difficult in clinical practice to distinguish pain symptoms from related irreversible skeletal complications such as arthropathy. When there is biochemical evidence of osteomalacia or insufficiency fractures, treatment may be of use. In all cases, treatment should be monitored carefully to prevent complications and avoid nephrocalcinosis. For further review of treatment options, please refer to the excellent review article: A clinician’s guide to X-linked hypophosphatemia [13]. A new treatment for both XLH and tumor-induced osteomalacia with high FGF23 levels is the administration of an Anti-FGF23 Antibody (Burosumab, Crysvita®, Ultragenyx). Recent results from a phase 2 and phase 3 studies have shown that burosumab can reduce the loss of phosphate from the kidney, improve abnormally low serum phosphate concentrations and reduces the severity of rickets as shown in x-rays [15, 16]. Treatment has recently received marketing approval in the EU for children with XLH and is being reviewed in the US for both pediatric an adult indications.

### Lysosomal storage disorders

Primary involvement of the bone marrow in inborn errors of metabolism is mainly found in the sphingolipidoses, particularly in Niemann Pick diseases and Gaucher disease. In Gaucher disease, severe bone marrow infiltration with lipid-laden macrophages can occur. Gaucher disease is caused by deficient activity of the lysosomal enzyme β-glucocerebrosidase (GBA; EC 3.2.1.45). GBA hydrolyzes the natural glycosphingolipid glucocerebroside (or glucosylceramide; GC) into glucose and ceramide. Storage of GC in macrophages gives rise to hepatosplenomegaly and involvement of the bone marrow. Severe skeletal pathology can occur in this disorder when left untreated [17]. The pathophysiology involves mass effect of storage with cortical thinning, necrosis, fibrosis and probably low-grade inflammation. Osteopenia or osteoporosis is frequent. Timely intervention with intravenous enzyme replacement therapy or oral substrate inhibitors can prevent skeletal complications. Frequently, bone marrow examination is the clue to a diagnosis of Gaucher disease, since most patients present with splenomegalie and cytopenia, mimicking lymphoma or leukemia. Also in Niemann Pick disease, caused by acid sphingomyelinase deficiency (types A and B), skeletal features can be present as a consequence of storage cells in the marrow. This disorder mainly presents with hepatosplenomegaly and interstitial lung disease, and, in severe cases with neurological symptoms [18]. Bone marrow involvement is less prominent than observed in Gaucher disease. Other skeletal manifestations of the disease include joint and bone pain, decreased bone mineral density and osteoporosis with a risk of fragility fractures [19]. In young adults, short stature and delayed onset of puberty may occur, but most patients eventually attain a normal adult height [20]. Of note, so called “pseudo-Gaucher cells” can be found in a number of disorders and should not be mistaken for the disorders mentioned above: proper diagnostic tests should be employed including genetic and enzymatic tests to confirm any disorder presenting with marrow storage cells.

### The mucopolysaccharidoses and mucolipidoses

Skeletal abnormalities are the hallmark of lysosomal storage disorders caused by accumulation of glycosaminoglycans. There are eleven types of mucopolysaccharidosis (MPS; type I, II, III (with subtypes A,B,C and D), IV (subtypes A en B), VI, VII and IX), each caused by deficiency of one of 11 different GAG degrading enzymes [21, 22]. These are multi-systemic disorders, with a wide range in presence and severity of the different clinical features. Skeletal involvement however, is universal in these disorders (with the exception of MPS III) and is of very early onset. Radiological studies in young children with MPS I, II and VI show abnormal bone development [23] and a histological study of a MPS IV foetus show prenatal accumulation of GAGs in chondrocytes [24].

Virtually all bones can be affected in MPS, but alterations in the shape of the skull, the bones of hips, hands, feet and spinal column are most prominent [23]. The term most commonly used for the combination of developmental skeletal alterations in MPS is dysostosis multiplex. Length growth in MPS is reduced and the spinal abnormalities and hip dysplasia results in altered posture. Range of motion of the spine, hips, knees, shoulders, ankle and often also the elbows is reduced to variable degree in all MPS types [25, 26] apart from type IVA, where there is joint laxity, often associated with (sub) luxation of joints [27]. During life, the use of these abnormally shaped joint bones leads to progressive joint damage and secondary arthritic changes, limiting mobility [28, 29]. Hip surgery and later on hip replacement are often performed in order to limit pain and to try to maintain walking ability [28, 29]. Skeletal Pain is a frequent compliant in MPS patients [30]. Other complications of skeletal disease in the MPSes are restrictive pulmonary disease due to altered thorax shape and thoracic wall stiffness and myelum compression resulting from spinal abnormalities.

How GAG accumulation leads to abnormal skeletal development and the occurrence of (secondary) osteoarthritis is only partially understood. In the growing skeleton, the accumulation of GAGs affects growth plate functioning, as can be seen from the disturbed growth plate architecture in MPS mouse models and a limited number of human bone specimens [31]. Joint inflammation is apparent in MPS hips and knees and is most likely due to secondary to damage from movement of abnormally developed and maligned joints and a direct effect of increasing GAG accumulation, potentially mediated through activation of the Toll Like Receptor 4 [32, 33].

Mucolipidosis type II and III α/β or γ are disorders caused by deficiency of the enzyme UDP-N-acetyl glucosamine-1-phosphotransferase (GLcNAc-PTase), which is involved in phosphorylation of lysosomal enzymes, ensuring correct targeting to the lysosome. Aberrant targeting of these degradation enzymes results in lysosomal accumulation of their substrates, including glycosaminoglycans and (glyco)sphingolipids. Mucolipidosis type II is the most severe form, in which rapidly progressive airway, cardiac, skeletal and nervous system disease results in death in early childhood. Mucolipidosis type III has a broader phenotypic range, with milder affected patients surviving into adulthood and displaying primarily debilitating skeletal symptoms. Skeletal symptoms in mucolipidosis bear great similarities to those of mucopolysaccharidosis, with dysostosis multiplex, secondary osteoarthritis of the joints and complications such as myelum compression and severe arthrosis of (amongst others) the hip joints, requiring surgical interventions at an early age [28].

Treatment in the form of enzyme replacement therapy (ERT) is available for MPS type I, II, IV, VI. There may be some positive effect of ERT on joint mobility (specifically range of motion of the shoulder and hips) but by and large this effect is limited and even early treated patients go on to develop severe skeletal complications [34, 35]. Hematological stem cell transplantation, performed in MPS I to halt neurodegeneration, does not have a significant effect on the occurrence of skeletal symptoms either [36]. A small, short duration trial with an anti-inflammatory drug, pentosan polysulphate in MPS I suggests a positive effect on pain [37]. There are no disease specific therapies for mucolipidosis type II and III. In all these disorders supportive care is of great importance, focusing on pain management, physiotherapy and well timed surgical interventions executed by a team experienced in treating these complex multisystem disorders.

### Mannosidoses

Alpha mannosidosis is a lysosomal storage disorder in which deficiency of the enzyme α-mannosidase leads to accumulation of mannose-rich oligosaccharides. Similar to the mucopolysaccharidoses and mucolipidoses, this is a clinically widely variable multisystemic disorder with neurocognitive and psychiatric symptoms, corneal clouding or cataract, hearing loss, immune deficiencies, myopathy and skeletal abnormalities. Dysostosis multiplex with skull abnormalities, kyphoscoliosis, pectus carinatum, hip dysplasia and hand and feet deformities are present in variable degree in the majority of patients [38, 39]. Secondary osteoarthritis of the large joints can occur [40] and an increasing incidence of arthrosis is found as patients get older [41]. Alpha mannosidosis has been treated with hematopoeitic stem cell transplantation [42, 43], but there are not enough data to evaluate the effect on the musculoskeletal symptoms of the disorder. Enzyme replacement therapy has been recently granted marketing authorization under exceptional circumstances in the EU. Long term the data on the effectiveness of this treatment, including the effect on musculoskeletal symptoms, will be evaluated.

Beta mannosidosis is an extremely rare disorder caused by accumulation of complex disaccharides due to deficiency of the lysosomal beta-mannosidase enzyme, with few published cases. It has a wide clinical spectrum of which skeletal abnormalities can be a feature [44].

### Pycnodysostosis

Pycnodysostosis is an autosomal recessive metabolic bone disorder caused by mutations in the cathepsin K (CTSK) gene [45]. Cathepsin K is a lysosomal protease which is secreted into the sealed extracellular compartment, where it is involved in degradation of bone matrix proteins (e.g. collagen type I, osteonectin and osteopontin). Deficient cathepsin K activity results fragile bones with increased mineral content. Clinically, the disorder is characterized by short stature, pathological fractures and bone dysplasia [46, 47]. Especially craniofacial and dental abnormalities and stubby hands and feet with acroosteolysis are typical of pycnodysostosis, though the latter is not found in all patients [47–49]. No specific treatment for pycnodysostosis is available. Multidisciplinary supportive care, including attention for the risk of upper airway obstruction, is indicated [50].

### Alkaptonuria

Alkaptonuria is an autosomal recessive metabolic disorder in the catabolic pathway of the amino acids phenylalanine and tyrosine. Decreased activity of the enzyme homogentisate 1,2-dioxygenase leads to accumulation of homogentisic acid (HGA) and its oxydated derivative benzoquinone acetic acid (BQA) in connective tissue, cartilage and body fluids (e.g. urine, sweat). The deposition of BQA polymer in connective tissue triggers the progressive tissue damage observed in this disorder. The disorder is sometimes recognized early in life because of discoloration of urine when exposed to oxygen or alkali, but symptomatic disease generally does not develop until the third decade of life. Back and joint pain are the first symptoms to occur and over time, progressive disease results in progressive deformation of the spine and arthrosis of the large joints, necessitating joint replacement at an early age [51, 52]. Other complications of the disease are the formation of stones in the genitourinary tract and cardiac valve disease.

Limiting HGA production can be attempted by a protein restricted diet, though this is hard to comply with, especially in a period of life when individuals are still asymptomatic [53]. Nitisinone (2-(2-nitro-4-trifluoromethylbenzoyl)-1,3-cyclohexanedione, NTBC), inhibiting the enzyme 4-hydroxyphenylpyruvate dioxygenase, can be used to reduce HGA synthesis. In a first clinical treatment trial, urinary HGA excretion was reduced by >95% in response to the highest administered daily dose (8 mg) [54]. Safety of this treatment (NTBC increases tyrosine, with unknown long term consequences) and its effectiveness in preventing clinical complications remains to be proven and a phase 3 trial (Suitability of Nitisinone in Alkaptonuria 2 (SONIA 2)) is currently ongoing.

## Secondary involvement of the skeleton

Several inherited metabolic disorders have been reported to be associated with reduced bone density (summarized in Table 2). Factors contributing to the reduced mineralization in these disorders can be the disease itself (e.g. lysinuric protein intolerance), dietary treatment (all inborn errors of protein metabolism), hypogonadism (e.g. galactosemia) and/or low vitamin D status due to reduced sunlight exposure(e.g. erythropoietic protoporphyria).

### Galactosemia

In classic galactosemia, deficiency of the galactose-1-phosphate uridyltransferase enzyme results in accumulation of galactose and its derivatives, from both exogenous ingestion and endogenous production. Clinically, this results in life threatening liver disease, often complicated by E Coli sepsis, very early in life. Treatment with a lactose free, galactose restricted diet reverses (or in case of newborn screening prevents) the severe early illness, but long term complications such as ovarian failure and neurocognitive deficits still occur [55]. Bone density has been reported to be below the average values for the general population in several studies in children and adults with classic galactosemia, included in the 2017 meta-analysis by van Erven et al. [56]. This meta-analysis showed that bone mineral density was within two standard deviation of the mean in the majority of patients. An estimated 10–25% had a clinically significant reduced bone mass (Z score ≤ −2 SD). Whether this results in an increased fracture risk is unknown [56].

Factors contributing to low bone density in classic galactosemia are reduced dietary calcium and vitamin D intake, ovarian insufficiency, reduced physical activity in some patients and potentially the metabolic disorder itself [57, 58]. Treatment with calcium and vitamin D supplementation, and estrogen in patients with early ovarian insufficiency, improves bone mass in classic galactosemia patients [59, 60]. The additional effect of aging on bone mineral density and fractures remains to be established as early treated patients have not yet reached late adulthood.

### Homocystinuria

Classic homocystinuria is caused by a deficiency of the enzyme cystathionine beta-synthetase (CBS), leading to grossly elevated levels of homocysteine and methionine. Symptoms include lens dislocation, thromboembolisms and cognitive impairment. In bone, high levels of homocysteine impairs collagen type I crosslinking, resulting in reduced bone strength [61]. Patients have no overt skeletal abnormalities at birth, but untreated they develop skeletal abnormalities such as genu valgum, pes cavus and excess growth of long bones as well as osteopenia or osteoporosis [62, 63]. Vertebral fractures and spine deformities can occur. Treatment may consist of a methionine restricted diet and administration of pyridoxine and betaine, depending on whether or not a patient is pyridoxine responsive [64]. Treatment from an early age, with resultant lower homocysteine levels, prevents the occurrence of bone complications [65, 66].

### Glycogen storage disorders

This group of inborn errors are characterized by reduced glycogen degradation and or gluconeogenesis. There over 12 different types of glycogen storage disorders (GSDs) with enzyme deficiencies in these pathways. Dependent on where the enzymes are expressed, deficiency leads to a liver defect (hepatomegaly, hypoglycemia, lactate acidosis), muscle defect (exercise intolerance, myopathy) or a combination of both. In GSD I type a and b additional symptoms such as renal tubulopathy (1a and b) neutropenia and neutrophil and autoimmune phenomena (1b) occur. Low bone density has been described in GSD I type a and b, GSD II, GSD III, GSD V and GSD IX [67–70] 1995 [71–73].

In GSD type 1, factors contributing to the reduction in bone mineral density are poor metabolic control [71, 74, 75] and possibly the treatment with gCSF in GSD1b [74], though the latter relationship was not found in all studies [76]. In a recent study in 4 male GSD I (a and b) patients, all had either osteoporosis or osteopenia, suffered from hypogonadotropic hypogonadism and one patient had a spinal compression fracture at a very young age [75].

In GSD II (Pompe disease) a high incidence of osteoporosis, as well as (asymptomatic) vertebral fractures has been observed [68, 69]. Contributing factors to altered bone composition in Pompe disease are reduced muscle strength [68], lack of physical activity, but there may be a direct effect of the storage disease on bone metabolism as well [69].

### Phenylketonuria

Phenylketonuria (PKU), one of the most prevalent inborn errors of metabolism, is caused by the inability to adequately degrade the amino acid phenylalanine, due to a deficiency of the enzyme phenylalanine hydroxylase. Phenylalanine is neurotoxic and its accumulation causes progressive neurological damage. Since the 1960s newborn screening for PKU is implemented and treatment with a protein restricted diet, combined with phenylalanine free amino acid supplementation, results in preservation of neurocognitive development [77]. A meta- analysis of all publications up to 2015 on bone density in PKU indicated that bone density is on average lower in PKU patients compared to the control population, but not in the range of osteoporosis or osteopenia [78]. Contributing factors to the reduced bone mineral density may be the low protein diet (despite amino acid supplementation) and/or the elevated phenylalanine levels. Low calcium, vitamin D or other vitamins and micronutrient intake is unlikely to influence bone density in the current PKU population, since these are sufficiently present in the presently used amino acid supplements. There is currently no conclusive evidence indicating a higher fracture risk in adults with early treated PKU [79, 80]. Since worldwide newborn screening for PKU has been introduced from the late 1960ties onwards, the effect of aging on fracture rate in early PKU patients cannot yet be established.

### Urea cycle defects, organic acidurias and lysinuric protein intolerance

Urea cycle disorders (UCDs) are a group of inborn errors in which conversion of nitrogen, from either dietary protein or body protein catabolism, to urea is disturbed, causing an increase in neurotoxic ammonia. Clinically, these disorders are characterized by episodes with progressive symptomology of lethargy, anorexia, altered behavior, drowsiness, epilepsy and ultimately coma. Organic acidurias are caused by specific enzyme deficiencies in the intermediary pathways of carbohydrate, protein and fatty acid metabolism, leading to the production of large amount of organic acids. The phenotypical spectrum of these disorder is broader compared to urea cycle disorder and apart may include for example renal, hematological and cardiac complications in addition to the neurocognitive manifestations. As in urea cycle disorders, acute metabolic decompensation, characterized by a high anion gap metabolic acidosis and/or hyperammonemia, may occur as a result of a catabolic state or high protein intake. Treatment of UCDs and organic acidurias, dependent on the type and severity of the disorder, exists of a protein restricted diet, avoidance of catabolic state and ammonia scavengers.

For the urea cycle disorders and organic acidurias the composition of the therapeutic low protein diet and/or the disorders itself may cause low bone mineral density, but limited data on bone density and factors influencing bone density are available for these conditions. In a case series of 81 methylmalonic and propionic aciduria patients, the bone density Z score was low in the majority of patients [81]. In another eight adult organic aciduria patients studied, osteoporosis was present in 2 and osteopenia in 4 patients, with one propionic aciduria patient suffering spinal fractures [82]. In a very small study in male urea cycle defect patients, bone mineral density was lower compared to aged matched control subjects [83].

Lysinuric protein intolerance (LPI), a condition in which reduced activity of the cationic amino acid transporter SLC7A7 leads to reduced intestinal uptake and increased urinary loss of amino acids, is associated with frank osteoporosis and an increased fracture rate [84]. Fractures may even be the presenting symptom in LPI [85, 86]. Classic symptoms of this disorder are intolerance for protein rich food, symptomatic hyperammonemia, failure to thrive. Long term complications include pulmonary alveolar and progressive renal disease. Reduced amino acid availability, resulting from the low protein content of diet and the deficient cationic amino acid transporter activity, may lead to reduced bone density through a decreased collagen synthesis [87]. A study in healthy discordant monozygotic twins suggest that specifically lower alanine and glycine intake are associated with lower bone mineral density [88]. Treatment of LPI consist of protein restriction, citrulline and/or lysine supplementation and nitrogen scavenger drugs [89]. Whether the pharmacological treatment, either through a direct effect of citrulline and/or lysine availability or through improvement in protein tolerance, has a positive effect on bone density and reduces fracture rate is currently unclear. Individual patients have also been treated with bisphosphonate therapy [90].

### Erythropoietic protoporphyria

In this inborn error of the haem biosynthesis, resulting from either ferochlatase deficiency or increased ALAS2 activity, protopophyrin IX accumulates in skin and liver. Protoporphyrin IX reacts with light, damaging skin endothelium by reactive oxygen species formation Clinically this manifests as first severe pain, and second, swelling and eryhthema of the skin if light exposure continues. As a result, patients avoid exposure to high intensity light, which in practice means they lead their life mainly indoors. This in turn leads to vitamin D deficiency and reduced physical activity, causing osteopenia and osteoporosis in a significant proportion of patients [91, 92].

### Wilson disease

In this autosomal recessive disorder, copper accumulation occurs due to mutations in the ATP7B gene, encoding for a copper transporting ATPase. The disease is characterized by hepatic, neurological and psychiatric manifestations. Osteoporosis as well as osteopenia occurs more frequently in adult patients compared to control subjects [93] and may be associated with a higher fracture rate [94]. Early onset osteoarthritis of the large joints (predominantly the knees) as well as lower back pain with spinal radiological abnormalities have been described in Wilson patients [95, 96].

## Diagnosis: When to suspect an inborn error of metabolism

In this section, diagnostic clues to uncover an underlying inborn error of metabolism are discussed for patients presenting with skeletal symptoms. Hence this refers to the first group discussed in this article: those with primary involvement of the skeleton. In clinical practice, patients will be referred to a specialist when they have skeletal deformities, retarded growth or both. This is particularly the case for children. In adults, more frequently subtle deformities, pain or fractures can be initial symptoms of an underlying metabolic defect. Hence, in some attenuated phenotypes, the diagnostic delay can be very long. In the current era of rapidly expanding genetic screening, whole exome sequencing may occur nowadays at an earlier stage. However, these techniques are not readily available in every center. In addition, variants of unknown significance may be discovered, which implicates that a combined approach, using important clinical, biochemical, radiological and other tools are needed for a correct diagnosis [97]. To improve awareness for metabolic disorders amongst the high number of acquired and inherited skeletal dysplasias and other factors that may affect bone, a brief summary of clues to a diagnosis of inborn errors of metabolism will be discussed (summarized in Tables 1 and 2).

### Additional clinical signs or symptoms

Within the group of lysosomal storage disorders, Gaucher disease is an example of a disorder that may present with pain as a first symptom. Although in most cases splenomegaly or cytopenia are the first symptom, in rare instances patients may come to medical attention because of a skeletal problem. Severe bone crises may occur, as well as avascular necrosis of the femoral head [17]. Additional signs are typical radiographic images such as Erlenmeyer flask deformities of the femurs. Both in Gaucher disease and pycnodysostosis, pathological fractures are reported. In acid sphingomyelinase deficiency, skeletal disease is much less prominent, but arthropathy may occur. The mucopolysaccharidoses have as most prominent features the dysplasia of the skeleton, but may also also present as a first sign in attenuated phenotypes with femoral head abnormalities resembling avascular necrosis [98]). True avascular necrosis of the femoral head is more frequent in MPS III, Sanfilipo disease [99]. Early arthropathy is common, and is also the main symptom of skeletal disease in the mucolipidoses. Joint stiffness and carpal tunnel syndrome are frequent symptoms of ML III, which resembles features of MPS I and VI. In mild MPS cases, suchs as MPSI-Scheie, arthropathy of the hands may mimick rheumatological disease [100]. Arthropathy is also common in alkaptonuria and hypophosphatasia. In the latter, chronic pain may also be the result of myopathy [4]. Abnormalities of the spine can be found in Gaucher disease (pathological fractures, crises, kyphosis) and in MPS disorders (dysplasia, kypohosis, scoliosis). Osteoporosis is rarely an isolated finding and unlikely to be a presenting symptom.

Thus, in patients with joint stiffness, early arthropathy, rheumatoid factor negative arthritis, femoral head necrosis, unexplained bone pain, radiographical evidence of marrow expansion or skeletal dysplasia, especially in the presence of other features such as abnormal growth, underlying metabolic disorders should be part of the differential diagnosis.

### Biochemistry

The presence of very low levels of bone-specific alkaline phosphatase and calcific deposits are a clue to the diagnosis of hypophosphatasia [11]. In hereditary hypophosphatemic rickets hypophosphatemia and low-normal circulating 1,25-dihydroxyvitamin D [1,25(OH)2D] levels are typical biochemical findings. Serum alkaline phosphatase (ALP) activity can be elevated in children and also sometimes in adult patients. Especially in patients with mild phenotypes, a low 25-vitamin D level with elevated ALP will be easily mistaken for vitamin D deficiency. The low phosphate level is the clue to the diagnosis in this case. In all other metabolic disorders presenting with skeletal features, routine biochemistry is usually normal.

### Genetics

In addition to the diagnostic clues described above, appropriate genetic testing should always follow for confirmation of the diagnosis, although in some cases, enzymatic testing is the gold standard for diagnosis (e.g. in the lysosomal storage disorders). Genetic counseling is essential to find other affected family members, assist in decision making for family planning and in some cases to be able to predict disease outcome.

## Therapy and management

Skeletal symptoms, in the form of dysplasia, arthropathy and/or reduced mineral density are part of the symptomology of a large number of inborn errors of metabolism. Due to better targeted therapies, as well as supportive care, more patients survive into adulthood and in many cases these skeletal symptoms become a more prominent feature of these disorders. This makes awareness of these skeletal symptoms as well as the options for management of the accompanying complaints all the more important.

Factors positively influencing healthy bone formation in the general population are adequate intake of calcium and vitamin D, preventing underweight, avoiding tobacco and excessive alcohol use and performance of weight bearing exercise. Their importance needs to be emphasized in the care of patients suffering from IEMs, in whom some of these aspects may provide a challenge. In case of primary ovarian failure (galactosemia) or hypogonadism (e.g. in glycogen storage disorder type I) appropriate hormone replacement therapy should be started to prevent osteopenia or osteoporosis. The role of bisphosphonate therapy in the treatment of patients with IEMs is not well established and currently the best advice is to treat osteopenia and/or osteoporosis according to general guidelines. In hereditary hypophosphatemic rickets treatment with active vitamin D and phosphate in childhood is important.

For several disorders, disease specific therapy with an effect on bone metabolism is or will become available, e.g. Asfotase alfa for hypophosphatasia, anti-IGF23 for hypophosphatemic rickets and Nitisinone for alkaptonuria. The long term effect of these drug on the skeletal manifestation of these disorders remains to be established. Pain management, physiotherapy and well timed surgical interventions, executed by a team experienced in treating these complex multisystem disorders, remain the cornerstone of disease management and their importance in maintaining mobility and improving quality of life should not be underestimated.

## References

[1] Dussault PM, Lazzari AA (2017). Epilepsy and osteoporosis risk. Curr Opin Endocrinol Diabetes Obes.

[2] Gattineni J (2014). Inherited disorders of calcium and phosphate metabolism. Curr Opin Pediatr.

[3] Linglart A, Biosse-Duplan M (2016). Hypophosphatasia. Curr Osteoporos Rep.

[4] Fonta C, Salles JP (2017). Neuromuscular features of hypophosphatasia. Arch Pediatr.

[5] Conti F, Ciullini L, Pugliese G (2017). Hypophosphatasia: clinical manifestation and burden of disease in adult patients. Clin Cases Miner Bone Metab.

[6] Whyte MP, Mumm S, Deal C (2007). Adult hypophosphatasia treated with teriparatide. J Clin Endocrinol Metab.

[7] Whyte MP, Greenberg CR, Salman NJ, Bober MB, McAlister WH, Wenkert D (2012). Enzyme-replacement therapy in life-threatening hypophosphatasia. N Engl J Med.

[8] Whyte MP, Madson KL, Phillips D, Reeves AL, McAlister WH, Yakimoski A (2016). Asfotase alfa therapy for children with hypophosphatasia. JCI Insight.

[9] Whyte Michael P., Rockman-Greenberg Cheryl, Ozono Keiichi, Riese Richard, Moseley Scott, Melian Agustin, Thompson David D., Bishop Nicholas, Hofmann Christine (2016). Asfotase Alfa Treatment Improves Survival for Perinatal and Infantile Hypophosphatasia. The Journal of Clinical Endocrinology & Metabolism.

[10] Costain Gregory, Moore Aideen M., Munroe Lauren, Williams Alison, Zlotnik Shaul Randi, Rockman-Greenberg Cheryl, Offringa Martin, Kannu Peter (2018). Enzyme replacement therapy in perinatal hypophosphatasia: Case report of a negative outcome and lessons for clinical practice. Molecular Genetics and Metabolism Reports.

[11] Ruppe MD. X-Linked Hypophosphatemia. In: Adam MP, Ardinger HH, Pagon RA, Wallace SE, Bean LJH, Stephens K et al., editors. GeneReviews((R)). Seattle (WA) 1993. Available from http://www.ncbi.nlm.nih.gov/books/NBK83985/ PubMedPMID:22319799. Accessed 1 June 2018.

[12] Quinlan C, Guegan K, Offiah A, Neill RO, Hiorns MP, Ellard S (2012). Growth in PHEX-associated X-linked hypophosphatemic rickets: the importance of early treatment. Pediatr Nephrol.

[13] Carpenter Thomas O, Imel Erik A, Holm Ingrid A, Jan de Beur Suzanne M, Insogna Karl L (2011). A clinician's guide to X-linked hypophosphatemia. Journal of Bone and Mineral Research.

[14] Rothenbuhler Anya, Esterle Laure, Gueorguieva Iva, Salles Jean-Pierre, Mignot Brigitte, Colle Michel, Linglart Agnes (2017). Two-year recombinant human growth hormone (rhGH) treatment is more effective in pre-pubertal compared to pubertal short children with X-linked hypophosphatemic rickets (XLHR). Growth Hormone & IGF Research.

[15] Imel EA, Ruppe MD, Weber TJ, Klausner MA, Wooddell MM, Carpenter TO (2014). Randomized trial of the anti-FGF23 antibody KRN23 in X-linked hypophosphatemia. J Clin Invest.

[16] Strensiq EPAR: http://www.ema.europa.eu/docs/en_GB/document_library/EPAR_-_Public_assessment_report/human/003794/WC500194340.pdf.

[17] Mikosch P, Hughes D (2010). An overview on bone manifestations in Gaucher disease. Wien Med Wochenschr.

[18] Schuchman EH, Wasserstein MP (2016). Types a and B Niemann-pick disease. Pediatr Endocrinol Rev.

[19] Wasserstein M, Godbold J, McGovern MM (2013). Skeletal manifestations in pediatric and adult patients with Niemann pick disease type B. J Inherit Metab Dis.

[20] Wasserstein MP, Larkin AE, Glass RB, Schuchman EH, Desnick RJ, McGovern MM (2003). Growth restriction in children with type B Niemann-pick disease. J Pediatr.

[21] Muenzer J (2011). Overview of the mucopolysaccharidoses. Rheumatology (Oxford).

[22] Clarke LA (2008). The mucopolysaccharidoses: a success of molecular medicine. Expert Rev Mol Med.

[23] Palmucci S, Attina G, Lanza ML, Belfiore G, Cappello G, Foti PV (2013). Imaging findings of mucopolysaccharidoses: a pictorial review. Insights Imaging.

[24] Beck M, Braun S, Coerdt W, Merz E, Young E, Sewell AC (1992). Fetal presentation of Morquio disease type a. Prenat Diagn.

[25] Guarany NR, Schwartz IV, Guarany FC, Giugliani R (2012). Functional capacity evaluation of patients with mucopolysaccharidosis. J Pediatr Rehabil Med.

[26] Tylki-Szymanska A, Marucha J, Jurecka A, Syczewska M, Czartoryska B (2010). Efficacy of recombinant human alpha-L-iduronidase (laronidase) on restricted range of motion of upper extremities in mucopolysaccharidosis type I patients. J Inherit Metab Dis.

[27] Tomatsu S, Yasuda E, Patel P, Ruhnke K, Shimada T, Mackenzie WG (2014). Morquio a syndrome: diagnosis and current and future therapies. Pediatr Endocrinol Rev.

[28] Oussoren E, Bessems J, Pollet V, van der Meijden JC, van der Giessen LJ, Plug I (2017). A long term follow-up study of the development of hip disease in Mucopolysaccharidosis type VI. Mol Genet Metab.

[29] Langereis Eveline J., den Os Matthijs M., Breen Catherine, Jones Simon A., Knaven Olga C., Mercer Jean, Miller Weston P., Kelly Paula M., Kennedy Jim, Ketterl Tyler G., O’Meara Anne, Orchard Paul J., Lund Troy C., van Rijn Rick R., Sakkers Ralph J., White Klane K., Wijburg Frits A. (2016). Progression of Hip Dysplasia in Mucopolysaccharidosis Type I Hurler After Successful Hematopoietic Stem Cell Transplantation. The Journal of Bone and Joint Surgery.

[30] Brands MM, Gungor D, van den Hout JM, Karstens FP, Oussoren E, Plug I (2015). Pain: a prevalent feature in patients with mucopolysaccharidosis. Results of a cross-sectional national survey. J Inherit Metab Dis.

[31] Clarke LA, Hollak CE (2015). The clinical spectrum and pathophysiology of skeletal complications in lysosomal storage disorders. Best Pract Res Clin Endocrinol Metab.

[32] Oussoren E, Brands MM, Ruijter GJ, der Ploeg AT, Reuser AJ (2011). Bone, joint and tooth development in mucopolysaccharidoses: relevance to therapeutic options. Biochim Biophys Acta.

[33] Opoka-Winiarska V, Jurecka A, Emeryk A, Tylki-Szymanska A (2013). Osteoimmunology in mucopolysaccharidoses type I, II, VI and VII. Immunological regulation of the osteoarticular system in the course of metabolic inflammation. Osteoarthr Cartil.

[34] Tomatsu S, Almeciga-Diaz CJ, Montano AM, Yabe H, Tanaka A, Dung VC (2015). Therapies for the bone in mucopolysaccharidoses. Mol Genet Metab.

[35] Muenzer J (2014). Early initiation of enzyme replacement therapy for the mucopolysaccharidoses. Mol Genet Metab.

[36] van der Linden MH, Kruyt MC, Sakkers RJ, de Koning TJ, Oner FC, Castelein RM (2011). Orthopaedic management of Hurler's disease after hematopoietic stem cell transplantation: a systematic review. J Inherit Metab Dis.

[37] Hennermann JB, Gokce S, Solyom A, Mengel E, Schuchman EH, Simonaro CM (2016). Treatment with pentosan polysulphate in patients with MPS I: results from an open label, randomized, monocentric phase II study. J Inherit Metab Dis.

[38] Spranger J, Gehler J, Cantz M (1976). The radiographic features of mannosidosis. Radiology.

[39] Beck Michael, Olsen Klaus J, Wraith James E, Zeman Jiri, Michalski Jean-Claude, Saftig Paul, Fogh Jens, Malm Dag (2013). Natural history of alpha mannosidosis a longitudinal study. Orphanet Journal of Rare Diseases.

[40] Gerards AH, Winia WP, Westerga J, Dijkmans BA, van Soesbergen RM (2004). Destructive joint disease in alpha-mannosidosis. A case report and review of the literature. Clin Rheumatol.

[41] Malm D, Riise Stensland HM, Edvardsen O, Nilssen O (2014). The natural course and complications of alpha-mannosidosis--a retrospective and descriptive study. J Inherit Metab Dis.

[42] Grewal Satkiran S., Shapiro Elsa G., Krivit William, Charnas Lawrence, Lockman Lawrence A., Delaney Kathleen A., Davies Stella M., Wenger David A., Rimell Frank L., Abel Susan, Grovas Alfred C., Orchard Paul J., Wagner John E., Peters Charles (2004). Effective treatment of α-mannosidosis by allogeneic hematopoietic stem cell transplantation. The Journal of Pediatrics.

[43] Yesilipek AM, Akcan M, Karasu G, Uygun V, Kupesiz A, Hazar V (2012). Successful unrelated bone marrow transplantation in two siblings with alpha-mannosidosis. Pediatr Transplant.

[44] Bedilu R, Nummy KA, Cooper A, Wevers R, Smeitink J, Kleijer WJ, et al. Variable clinical presentation of lysosomal beta-mannosidosis in patients with null mutations. Mol Genet Metab. 2002;77(4):282–90. 10.1016/s1096-7192(02)00172-5 12468273

[45] Gelb BD, Shi GP, Chapman HA, Desnick RJ (1996). Pycnodysostosis, a lysosomal disease caused by cathepsin K deficiency. Science.

[46] Lamy M, Maroteaux P (1965). Pycnodysostosis. Rev Esp Pediatr.

[47] Xue Yang, Cai Tao, Shi Songtao, Wang Weiguang, Zhang Yanli, Mao Tianqiu, Duan Xiaohong (2011). Clinical and animal research findings in pycnodysostosis and gene mutations of cathepsin K from 1996 to 2011. Orphanet Journal of Rare Diseases.

[48] Arman A, Bereket A, Coker A, Kiper PO, Guran T, Ozkan B (2014). Cathepsin K analysis in a pycnodysostosis cohort: demographic, genotypic and phenotypic features. Orphanet J Rare Dis.

[49] Aghili H, Tabatabaei SMA, Goldani MM (2017). Clinical and radiographic features of Pycnodysostosis with emphasis on Dentofacial problems. Case Rep Dent.

[50] Testani Elisa, Scarano Emanuele, Leoni Chiara, Dittoni Serena, Losurdo Anna, Colicchio Salvatore, Gnoni Valentina, Vollono Catello, Zampino Giuseppe, Paludetti Gaetano, Marca Giacomo Della (2014). Upper airway surgery of obstructive sleep apnea in pycnodysostosis: Case report and literature review. American Journal of Medical Genetics Part A.

[51] Introne WJ, Gahl WA. Alkaptonuria. In: Adam MP, Ardinger HH, Pagon RA, Wallace SE, Bean LJH, Stephens K et al., editors. GeneReviews((R)). Seattle (WA) 1993. Available from http://www.ncbi.nlm.nih.gov/books/NBK1454/ PubMed PMID: 20301627. Accessed 1 June 2018.

[52] Ranganath LR, Cox TF (2011). Natural history of alkaptonuria revisited: analyses based on scoring systems. J Inherit Metab Dis.

[53] Arnoux JB, Le Quan Sang KH, Brassier A, Grisel C, Servais A, Wippf J (2015). Old treatments for new insights and strategies: proposed management in adults and children with alkaptonuria. J Inherit Metab Dis.

[54] Ranganath Lakshminarayan R, Milan Anna M, Hughes Andrew T, Dutton John J, Fitzgerald Richard, Briggs Michael C, Bygott Helen, Psarelli Eftychia E, Cox Trevor F, Gallagher James A, Jarvis Jonathan C, van Kan Christa, Hall Anthony K, Laan Dinny, Olsson Birgitta, Szamosi Johan, Rudebeck Mattias, Kullenberg Torbjörn, Cronlund Arvid, Svensson Lennart, Junestrand Carin, Ayoob Hana, Timmis Oliver G, Sireau Nicolas, Le Quan Sang Kim-Hanh, Genovese Federica, Braconi Daniela, Santucci Annalisa, Nemethova Martina, Zatkova Andrea, McCaffrey Judith, Christensen Peter, Ross Gordon, Imrich Richard, Rovensky Jozef (2014). Suitability Of Nitisinone In Alkaptonuria 1 (SONIA 1): an international, multicentre, randomised, open-label, no-treatment controlled, parallel-group, dose-response study to investigate the effect of once daily nitisinone on 24-h urinary homogentisic acid excretion in patients with alkaptonuria after 4 weeks of treatment. Annals of the Rheumatic Diseases.

[55] Coelho AI, Rubio-Gozalbo ME, Vicente JB, Rivera I (2017). Sweet and sour: an update on classic galactosemia. J Inherit Metab Dis.

[56] van Erven B, Welling L, van Calcar SC, Doulgeraki A, Eyskens F, Gribben J (2017). Bone health in classic Galactosemia: systematic review and Meta-analysis. JIMD Rep.

[57] Batey L. A., Welt C. K., Rohr F., Wessel A., Anastasoaie V., Feldman H. A., Guo C.-Y., Rubio-Gozalbo E., Berry G., Gordon C. M. (2012). Skeletal health in adult patients with classic galactosemia. Osteoporosis International.

[58] Waisbren Susan E., Potter Nancy L., Gordon Catherine M., Green Robert C., Greenstein Patricia, Gubbels Cynthia S., Rubio-Gozalbo Estela, Schomer Donald, Welt Corrine, Anastasoaie Vera, D’Anna Kali, Gentile Jennifer, Guo Chao-Yu, Hecht Leah, Jackson Roberta, Jansma Bernadette M., Li Yijun, Lip Va, Miller David T., Murray Michael, Power Leslie, Quinn Nicolle, Rohr Frances, Shen Yiping, Skinder-Meredith Amy, Timmers Inge, Tunick Rachel, Wessel Ann, Wu Bai-Lin, Levy Harvey, Elsas Louis, Berry Gerard T. (2011). The adult galactosemic phenotype. Journal of Inherited Metabolic Disease.

[59] Panis B, Vermeer C, van Kroonenburgh M, Nieman FHM, Menheere P, Spaapen LJ (2006). Effect of calcium, vitamins K1 and D3 on bone in galactosemia. Bone.

[60] Fridovich-Keil JL, Gubbels CS, Spencer JB, Sanders RD, Land JA, Rubio-Gozalbo E (2011). Ovarian function in girls and women with GALT-deficiency galactosemia. J Inherit Metab Dis.

[61] Levasseur R (2009). Bone tissue and hyperhomocysteinemia. Joint Bone Spine.

[62] Mudd SH, Skovby F, Levy HL, Pettigrew KD, Wilcken B, Pyeritz RE, et al. The natural history of homocystinuria due to cystathionine beta-synthase deficiency. Am J Hum Genet. 1985;37(1):1–31. PMC1684548 3872065

[63] Weber David R., Coughlin Curtis, Brodsky Jill L., Lindstrom Kristin, Ficicioglu Can, Kaplan Paige, Freehauf Cynthia L., Levine Michael A. (2016). Low bone mineral density is a common finding in patients with homocystinuria. Molecular Genetics and Metabolism.

[64] Morris AA, Kozich V, Santra S, Andria G, Ben-Omran TI, Chakrapani AB (2017). Guidelines for the diagnosis and management of cystathionine beta-synthase deficiency. J Inherit Metab Dis.

[65] Lim JS, Lee DH (2013). Changes in bone mineral density and body composition of children with well-controlled homocystinuria caused by CBS deficiency. Osteoporos Int.

[66] Yap S, Naughten E (1998). Homocystinuria due to cystathionine beta-synthase deficiency in Ireland: 25 years' experience of a newborn screened and treated population with reference to clinical outcome and biochemical control. J Inherit Metab Dis.

[67] Mundy HR, Williams JE, Lee PJ, Fewtrell MS (2008). Reduction in bone mineral density in glycogenosis type III may be due to a mixed muscle and bone deficit. J Inherit Metab Dis.

[68] van den Berg LE, Zandbergen AA, van Capelle CI, de Vries JM, Hop WC, van den Hout JM (2010). Low bone mass in Pompe disease: muscular strength as a predictor of bone mineral density. Bone.

[69] Bertoldo Francesco, Zappini Francesca, Brigo Martina, Moggio Maurizio, Lucchini Valeria, Angelini Corrado, Semplicini Claudio, Filosto Massimiliano, Ravaglia Sabrina, Cotelli Sofia, Todeschini Alice, Scarpelli Mauro, Pancheri Serena, Tonin Paola (2015). Prevalence of Asymptomatic Vertebral Fractures in Late-Onset Pompe Disease. The Journal of Clinical Endocrinology & Metabolism.

[70] Lee PJ, Patel JS, Fewtrell M, Leonard JV, Bishop NJ (1995). Bone mineralisation in type 1 glycogen storage disease. Eur J Pediatr.

[71] Schwahn B, Rauch F, Wendel U, Schonau E (2002). Low bone mass in glycogen storage disease type 1 is associated with reduced muscle force and poor metabolic control. J Pediatr.

[72] Rake JP, Visser G, Huismans D, Huitema S, van der Veer E, Piers DA (2003). Bone mineral density in children, adolescents and adults with glycogen storage disease type Ia: a cross-sectional and longitudinal study. J Inherit Metab Dis.

[73] Rodriguez-Gomez I, Santalla A, Diez-Bermejo J, Munguia-Izquierdo D, Alegre LM, Nogales-Gadea G (2018). A new condition in McArdle disease: poor bone health-benefits of an active lifestyle. Med Sci Sports Exerc.

[74] Melis D, Pivonello R, Cozzolino M, Della Casa R, Balivo F, Del Puente A (2014). Impaired bone metabolism in glycogen storage disease type 1 is associated with poor metabolic control in type 1a and with granulocyte colony-stimulating factor therapy in type 1b. Horm Res Paediatr.

[75] Wong EM, Lehman A, Acott P, Gillis J, Metzger DL, Sirrs S (2017). Hypogonadotropic hypogonadism in males with glycogen storage disease type 1. JIMD Rep.

[76] Minarich LA, Kirpich A, Fiske LM, Weinstein DA (2012). Bone mineral density in glycogen storage disease type Ia and Ib. Genet Med.

[77] Al Hafid N, Christodoulou J (2015). Phenylketonuria: a review of current and future treatments. Transl Pediatr.

[78] Demirdas S, Coakley KE, Bisschop PH, Hollak CE, Bosch AM, Singh RH (2015). Bone health in phenylketonuria: a systematic review and meta-analysis. Orphanet J Rare Dis.

[79] Hansen KE, Ney D (2014). A systematic review of bone mineral density and fractures in phenylketonuria. J Inherit Metab Dis.

[80] Demirdas Serwet, van Spronsen Francjan J., Hollak Carla E.M., van der Lee J. Hanneke, Bisschop Peter H., Vaz Fred M., ter Horst Nienke M., Rubio-Gozalbo M. Estela, Bosch Annet M. (2017). Micronutrients, Essential Fatty Acids and Bone Health in Phenylketonuria. Annals of Nutrition and Metabolism.

[81] Touati G., Valayannopoulos V., Mention K., de Lonlay P., Jouvet P., Depondt E., Assoun M., Souberbielle J. C., Rabier D., Ogier de Baulny H., Saudubray J.-M. (2006). Methylmalonic and propionic acidurias: Management without or with a few supplements of specific amino acid mixture. Journal of Inherited Metabolic Disease.

[82] Martin-Hernandez E, Lee PJ, Micciche A, Grunewald S, Lachmann RH (2009). Long-term needs of adult patients with organic acidaemias: outcome and prognostic factors. J Inherit Metab Dis.

[83] Wilcox G, Strauss BJ, Francis DE, Upton H, Boneh A (2005). Body composition in young adults with inborn errors of protein metabolism--a pilot study. J Inherit Metab Dis.

[84] Simell O, Perheentupa J, Rapola J, Visakorpi JK, Eskelin LE (1975). Lysinuric protein intolerance. Am J Med.

[85] Posey Jennifer E., Burrage Lindsay C., Miller Marcus J., Liu Pengfei, Hardison Matthew T., Elsea Sarah H., Sun Qin, Yang Yaping, Willis Alecia S., Schlesinger Alan E., Bacino Carlos A., Lee Brendan H. (2014). Lysinuric protein intolerance presenting with multiple fractures. Molecular Genetics and Metabolism Reports.

[86] Carpenter TO, Levy HL, Holtrop ME, Shih VE, Anast CS (1985). Lysinuric protein intolerance presenting as childhood osteoporosis. Clinical and skeletal response to citrulline therapy. N Engl J Med.

[87] Parto K, Penttinen R, Paronen I, Pelliniemi L, Simell O (1993). Osteoporosis in lysinuric protein intolerance. J Inherit Metab Dis.

[88] Jennings A, MacGregor A, Spector T, Cassidy A (2016). Amino acid intakes are associated with bone mineral density and prevalence of low bone mass in women: evidence from discordant monozygotic twins. J Bone Miner Res.

[89] Sebastio G, Sperandeo MP, Andria G (2011). Lysinuric protein intolerance: reviewing concepts on a multisystem disease. Am J Med Genet C: Semin Med Genet.

[90] Gomez L, Garcia-Cazorla A, Gutierrez A, Artuch R, Varea V, Martin J (2006). Treatment of severe osteoporosis with alendronate in a patient with lysinuric protein intolerance. J Inherit Metab Dis.

[91] Biewenga M., Matawlie R.H.S., Friesema E.C.H., Koole-Lesuis H., Langeveld M., Wilson J.H.P., Langendonk J.G. (2017). Osteoporosis in patients with erythropoietic protoporphyria. British Journal of Dermatology.

[92] Allo G, del Carmen G-AM, Mendez M, De Salamanca RE, Martinez G, Hawkins F (2013). Bone mineral density and vitamin D levels in erythropoietic protoporphyria. Endocrine.

[93] Weiss KH, Van de Moortele M, Gotthardt DN, Pfeiffenberger J, Seessle J, Ullrich E (2015). Bone demineralisation in a large cohort of Wilson disease patients. J Inherit Metab Dis.

[94] Quemeneur AS, Trocello JM, Ea HK, Ostertag A, Leyendecker A, Duclos-Vallee JC (2014). Bone status and fractures in 85 adults with Wilson's disease. Osteoporos Int.

[95] Golding DN, Walshe JM (1977). Arthropathy of Wilson's disease. Study of clinical and radiological features in 32 patients. Ann Rheum Dis.

[96] Quemeneur AS, Trocello JM, Ea HK, Woimant F, Liote F (2011). Miscellaneous non-inflammatory musculoskeletal conditions. Musculoskeletal conditions associated with Wilson's disease. Best Pract Res Clin Rheumatol.

[97] van Karnebeek Clara D. M., Wortmann Saskia B., Tarailo-Graovac Maja, Langeveld Mirjam, Ferreira Carlos R., van de Kamp Jiddeke M., Hollak Carla E., Wasserman Wyeth W., Waterham Hans R., Wevers Ron A., Haack Tobias B., Wanders Ronald J.A., Boycott Kym M. (2018). The role of the clinician in the multi-omics era: are you ready?. Journal of Inherited Metabolic Disease.

[98] Williams N, Challoumas D, Ketteridge D, Cundy PJ, Eastwood DM (2017). The mucopolysaccharidoses: advances in medical care lead to challenges in orthopaedic surgical care. Bone Joint J.

[99] de Ruijter J, Maas M, Janssen A, Wijburg FA (2013). High prevalence of femoral head necrosis in Mucopolysaccharidosis type III (Sanfilippo disease): a national, observational, cross-sectional study. Mol Genet Metab.

[100] Cimaz R, Vijay S, Haase C, Coppa GV, Bruni S, Wraith E, et al. Attenuated type I mucopolysaccharidosis in the differential diagnosis of juvenile idiopathic arthritis: a series of 13 patients with Scheie syndrome. Clin Exp Rheumatol. 2006;24(2):196–202. 16762159

